# Applications of In-Cell NMR in Structural Biology and Drug Discovery

**DOI:** 10.3390/ijms20010139

**Published:** 2019-01-02

**Authors:** CongBao Kang

**Affiliations:** Experimental Therapeutics Centre, Agency for Science, Technology and Research (A*STAR), 31 Biopolis way, Nanos, #03-01, Singapore 138669, Singapore; cbkang@etc.a-star.edu.sg; Tel.: +65-6407-0602

**Keywords:** in-cell NMR, protein structure, protein dynamics, drug discovery, target engagement, protein modification

## Abstract

In-cell nuclear magnetic resonance (NMR) is a method to provide the structural information of a target at an atomic level under physiological conditions and a full view of the conformational changes of a protein caused by ligand binding, post-translational modifications or protein–protein interactions in living cells. Previous in-cell NMR studies have focused on proteins that were overexpressed in bacterial cells and isotopically labeled proteins injected into oocytes of *Xenopus laevis* or delivered into human cells. Applications of in-cell NMR in probing protein modifications, conformational changes and ligand bindings have been carried out in mammalian cells by monitoring isotopically labeled proteins overexpressed in living cells. The available protocols and successful examples encourage wide applications of this technique in different fields such as drug discovery. Despite the challenges in this method, progress has been made in recent years. In this review, applications of in-cell NMR are summarized. The successful applications of this method in mammalian and bacterial cells make it feasible to play important roles in drug discovery, especially in the step of target engagement.

## 1. Introduction

Solution nuclear magnetic resonance (NMR) [1], X-ray crystallography and cryogenic electron microscopy (cryo-EM) [2] are important tools for obtaining the structures of biomolecules at atomic resolution [3]. When diffracted crystals are available, X-ray crystallography is a robust way to obtain high-resolution structures of biomolecules [4]. In recent years, the rapid development of cryo-EM has made it possible to solve structures of biomolecule complexes with high molecular weight at a high resolution. For example, the structures of many difficult targets such as ion channels and membrane-bound enzyme complexes were obtained using cryo-EM [5,6]. Other methods, such as small-angle X-ray/neutron scattering (SAXS/SANS) [7], mass spectrometry [8] and chemical cross-linking [9] are also used to determine structures of protein complexes.

Solution NMR spectroscopy is able to investigate protein structures and dynamics under solution conditions because the targets can be studied in different buffers and at various temperatures [10]. Although it is still challenging to study protein structures with high molecular mass due to the signal overlap and sensitivity, NMR has been widely used in protein chemistry and drug discovery with the development of magnets, pulse programs [11,12,13], and different protein-labeling strategies [14,15,16]. Solution NMR spectroscopy has been used in various research topics, including protein–protein, protein–nucleotide complexes, and membrane proteins, to provide useful information in order to understand protein structure and function [17,18,19,20]. Both solid and solution NMR spectroscopies have been successfully used to probe the structures of membrane proteins, which are normally challenging to crystallize [21,22,23]. Many membrane proteins have been characterized using solution and solid-state NMR spectroscopy [24,25,26].

NMR spectroscopy is a powerful method that can be used in combination with other methods, such as X-ray, cryo-EM, bioinformatics and SAXS/SANS, providing different views on the structures and dynamics of biomolecules, and their functional complexes in solution [27,28,29,30,31]. It is well known that NMR data analysis is time consuming. Therefore, NMR can work with other methods to save a lot of time in data processing and analysis. Available web servers, such as structure prediction and protein–protein binding interface predictions, can also speed up NMR data analysis [32,33,34,35,36,37,38,39]. The most frequently used strategy is to combine available structures obtained by using X-ray, cryo-EM or homology models with dynamic and ligand binding information obtained by NMR, which provides a full view of the target function, ligand binding modes, and regulation mechanisms [27]. Successful examples can be seen in many studies [40,41,42,43], and will not be described here.

As NMR is a powerful tool for monitoring the environmental changes of atoms, it has been used for probing protein–protein and –ligand interactions. In addition, NMR active nuclei such as ^19^F and ^31^P can be incorporated to a protein, making ^19^F and ^31^P NMR possible in determining conformational changes of proteins induced by ligand binding or post-translational modifications [44,45,46,47,48]. In fragment-based drug discovery (FBDD), NMR is frequently used in identifying fragments with different binding affinities [49,50]. Proton-based NMR spectroscopies have been successfully used in this field. As hetero-nuclear NMR experiments can be used to monitor environmental changes of individual amino acid of a protein, NMR is then very useful in generating the structure-activity relationship of a compound in a drug discovery project [47,51]. The available access to different types of compound libraries such as ^19^F-labeled compound libraries makes NMR an important tool in drug discovery by identifying novel hits, confirming hits obtained from biochemical assays, mapping the ligand binding site, probing the druggability of a target protein, and determining the ligand binding mode [45,46,48,52,53,54,55].

With the accumulation of structures of biomolecules determined by different methods such as X-ray and Cryo-EM, interest has been focused on the correlation between structure and function of biomolecules. Therefore, the information obtained from structural biology has to be connected well with that obtained from cell biology and biochemistry. It is critical that the structure of a biomolecule is determined under a condition that is close to the physiological environment. NMR is the most efficient structural tool to achieve such requirements [56]. Research has been carried out to study the structures of proteins in living cells using NMR techniques, which leads to the concept of in-cell NMR [57]. This unique approach bridges the gap between structural techniques and cellular imaging techniques [58,59]. This technique is also applicable to solid-state NMR [60,61]. This review only summarizes recent progress in in-cell NMR using solution NMR spectroscopy and discusses the challenges and potential applications in drug discovery.

## 2. In-Cell NMR

In-cell NMR was proposed to study protein dynamics and structures in living cells [62], making this method unique to others used for structural analysis [57,63]. It is a non-invasive method to determine the structure of a target under the physiological conditions [64]. As the cells used in in-cell NMR are alive, intact and contain complete cellular compartments, the obtained information is therefore very useful in biology, as well as other fields, such as drug discovery. Although structural studies of membrane proteins in living cells are of great interest for in-cell NMR, this review will mainly focus on in-cell NMR studies of water-soluble proteins carried out using solution-state NMR [61,65].

### 2.1. Cells Used in In-Cell NMR

Different cells, including bacteria, yeast, oocyte and mammalian cells, are able to be used for in-cell NMR studies. The most frequently used cell line is *E. coli* (Table 1 and Table 2). The application of in-cell NMR in mammalian cells make it attractive in target engagement in drug discovery when the targets are related to human diseases. It will be ideal when in-cell NMR can be carried out in all types of cells, while experiments have to be performed to obtain suitable conditions for gaining high-quality NMR spectra.

### 2.2. Isotopic Incorporation

**Figure 1:**
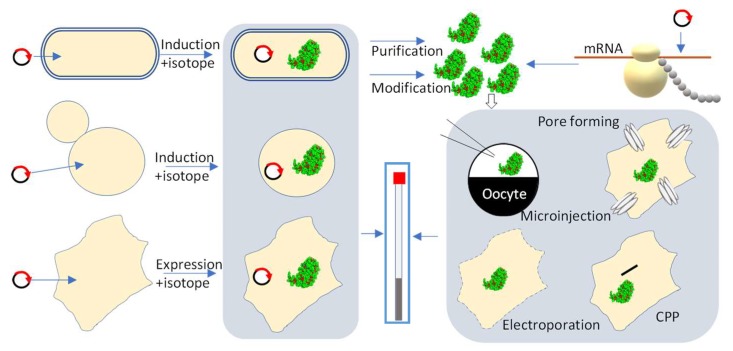
Sample preparation for in-cell NMR studies. The cells used for in-cell NMR studies can be prepared using the following strategies: Proteins (green) can be directly over-expressed in different cell lines using expression vectors. To make isotopically labeled proteins for in-cell NMR studies, the target gene can be cloned into suitable vectors followed with transfection/transformation into cells. Target protein can be isotopically labeled by growing cells in isotopically enriched (^15^N, ^15^N/^13^C) media. Cells with the overexpressed protein are then used for in-cell NMR experiments. Isotopically labeled proteins can also be prepared in vitro by overexpressing them in different cells or using cell-free expression systems. The labeled protein is then purified before being introduced to oocytes by microinjection. Blue box indicates the NMR tube. Labeled proteins can also be introduced into human cells using either cell-penetrating peptides (CPP), cell permeabilization by pore-forming toxins or electroporation as introduced previously [107]. This figure was modified from the figure of Luchinat and Banci [107].

Similar to the conventional NMR methods, to obtain high-quality in-cell NMR spectra, the proteins need to be isotopically labeled or contain NMR-active nuclei such as ^15^N and ^13^C. Labeling protein with ^19^F [105] or ^31^P is also a feasible strategy for in-cell NMR experiments, as ^19^F and ^31^P [106] NMR are commonly used in solution NMR studies. In-cell NMR has another advantage over other methods used for structural studies. Purifying the target protein is not required, which is very attractive for some targets that are difficult to prepare in vitro. Isotopically labeled proteins can be purified for in-cell NMR studies in mammalian cells, but they must be delivered to the cells (Figure 1) using cell-penetrating peptides, toxin microinjection, or electroporation methods [87,91,93]. Overexpressing the target proteins by growing cells in different medium is the most convenient way for in-cell NMR studies, which is achievable in both bacterial and mammalian cells (Table 2).

### 2.3. NMR Experiments for In-Cell NMR Studies

Although in-cell NMR experiments are similar to normal experiments that are carried out in vitro, several factors (below) will affect the selection of the experiments as challenges remain in the in-cell NMR studies. Normal one dimensional (1D) and multiple dimensional experiments can be collected (Table 1). As the available in-cell NMR studies focused on a few proteins (Table 2), more studies are needed to enlarge the application of this method.

### 2.4. Challenges in In-Cell NMR

Challenges remain for in-cell NMR in practice despite recent progress. Firstly, the target is present with other molecules in cells, which requires careful protein-labeling strategies to reduce the background signals. Secondly, the target protein might interact with other proteins to form complexes with high molecular weights which have rapid relaxation and low signal sensitivity. Unspecific interactions may also exist inside living cells, which will contribute to signal reduction in the NMR spectra. Optimized NMR pulse programs will be helpful in increasing the signal sensitivity and reducing data collection time [108,109]. Thirdly, injecting or delivering isotopically enriched proteins into cells is a good strategy for gaining signal intensity and reducing background noise, but the injected protein might be transported outside of the cells by different mechanisms. The leaky protein will exhibit signals influencing the in-cell NMR spectra [79]. When the protein is overexpressed in cells, the collapse of the dead cells will make the labeled protein released into the medium, which will give very sharp signals in the spectra [63,110]. A bioreactor in NMR tube can reduce cell death and make in-cell NMR sample last for a longer time [111,112]. Fourthly, the viscosity inside cells is higher than water, which can lead to line broadening of the signals [113,114]. Fifthly, the target protein may exist in different forms when it is over-expressed in the cells. The target protein might be in free form, in complexes with molecules and partially degraded by proteases. Such sample heterogeneity will give rise to in-cell NMR data with low quality. Sixthly, in ligand-binding studies, the tested ligands must be able to penetrate the cell membrane, which is different from the in vitro NMR study. The tested compounds should fulfill certain standards such as stability and cell penetrating activity when they are used in in-cell NMR experiments. Lastly, as in-cell NMR is monitoring spectra of a protein in living cells, the time required for data acquisition should be as short as possible because the target protein might be degraded by proteases. Strategies such as increasing protein stability, sustaining the life time of the cells, collecting data in a shorter time and using multiple samples in data collection will be helpful in in-cell NMR studies.

## 3. In-Cell NMR in Different Cells

In-cell NMR has been carried out in various cells (Table 2). Although most experiments are 1D and 2D types, accumulated studies (Table 3) provide evidence that other multiple dimensional experiments could be performed in various cell types.

### 3.1. In-Cell NMR in Bacterial Cells

In normal NMR samples, the concentration of the target protein is in the µM to mM range, with high purity (>90%). The concentration of a target protein in the living cells is normally very low, and there are a lot of proteins that might exhibit detectable NMR signals. The background signals from other molecules are very high if the cells are cultured in a medium containing isotopically labeled carbon and nitrogen sources. Overexpression of the target protein in the living cells is a strategy to gain signal intensities while the expression of other proteins should be properly suppressed.

To reduce the signal background of *E. coli* proteins, the following method can be used. The gene of a target protein cloned in an expression vector is first transformed into *E. coli* followed by culturing in the normal medium. Before the target protein was induced, the cultured bacterial cells were transferred into a medium containing isotopes [68], which reduced the background signals. This method was successfully used in the study of the putative heavy-metal binding protein TTHA1718. In the study, the sample was shown to be stable for 6 h. Backbone resonance assignment of the protein in cells were obtained using 3D experiments, which were collected using a nonlinear sampling scheme for the indirectly acquired dimensions [68]. In addition, selective protonation and ^13^C labeling of Ala, Leu and Val residues of the protein were obtained in *E. coli*, which made structural determination of TTHA1718 in *E. coli* possible. This study showed the structure of the protein in the living cells. Although the structure in vivo is similar to that determined in vitro, residues that interact with other proteins can be identified. Isotopic labeling of the protein can also be achieved by switching cells from unlabeled medium to an isotope enriched medium [78]. This method can also be used for labeling protein at the methyl groups [78].

Most proteins might not be suitable for in-cell NMR studies [118], which makes in-cell NMR in *E. coli* cells only applicable to some specific cases. In addition to TTHA1718, several proteins, such as NumerA [66], GB1, the N-terminal metal-binding domain of MerA [119] and human copper, zinc superoxide dismutase 1 (hSOD1) [72], were shown to exhibit nicely dispersed cross peaks in the spectra in in-cell NMR studies (Table 2). For the folded proteins, the difficulty in obtaining good quality NMR data is mainly due to crowding [120]. For mammalian proteins, *E. coli* might not be an ideal system for in-cell NMR studies and the mammalian cells should be considered [120]. In-cell NMR study on some intrinsically disordered proteins can be carried out in *E. coli* cells using an overexpression system [121]. The procedures for carrying out such experiments have been described in detail [121,88]. In-cell NMR in bacteria is a powerful tool to evaluate structure and dynamics of intrinsically disordered proteins [63,122,123]. Protein-based ^19^F-NMR was able to be carried out in *E. coli*, making it possible with this method to monitor proteins with high molecular weight [73]. Measuring the spin relaxation parameters was used to probe the interactions of intrinsically disordered protein and components of the cytosol in the living cells [74]. The dynamic parameters of intrinsically disordered proteins obtained using in-cell NMR under the physiological conditions will be useful for understanding their function and regulation [124].

### 3.2. In-Cell NMR in Yeast

Yeast cells such as *Pichia pastoris* are suitable for in-cell NMR studies, as they are used for overexpressing proteins in vitro NMR studies. For some mammalian proteins that are difficult to express in bacteria, yeast cells would be one option for protein production. In vitro NMR experiments demonstrated the interactions between ubiquitin and RNA in yeast [125]. Such interaction could be verified by in-cell NMR in yeast. A protocol for isotopic labeling of proteins in budding yeast was developed [90]. Ubiquitin was overexpressed using the *AOX1* promoter, which was induced by methanol. Ubiquitin in yeast cells was isotopically labeled and exhibited a dispersed NMR spectrum. The dynamic properties of ubiquitin in various cellular compartments, including cytosol and protein storage bodies, were explored using in-cell NMR. One advantage of using yeast in in-cell NMR studies is that the location of the overexpressed ubiquitin at different places were able to be achieved by growing cells in different growth media [90]. The impact of a target protein at different locations in living cells can therefore be investigated.

### 3.3. In-Cell NMR in Oocytes of Xenopus laevis

Oocyte was able to serve as a system for in-cell NMR studies in which microinjection of labeled proteins into the living cells was required [86]. As the size of the oocyte is larger than those of bacteria and mammalian cells, the amount of the cells in the NMR studies is less. Approximately 200 oocytes would be sufficient for one NMR measurement [87]. The cellular environment of the oocyte is close to that of the mammalian cells, which makes it a useful system to explore structure and function of human proteins [126,127]. To carry out in-cell NMR studies in oocytes, the target protein needs to be isotopically labeled, purified and then introduced into cells by microinjection. Several examples have proven the feasibility of this method. In a study carried out by Sakai et al., ^1^H-^15^N-HSQC spectrum of ubiquitin was obtained. Slightly different spectra of ubiquitin in cells and in vitro were observed. The amino acids that exhibited different chemical shifts in the spectra might be due to unspecific protein–protein interactions. In addition, maturation of ubiquitin precursor in the living cells was observed [86]. NMR studies of GB1 were also able to be carried out in oocytes [87]. In this study, purified GB1 was shown not to interact with any components of *Xenopus* egg extracts. The impact of BSA on the NMR spectra of GB1 was also investigated, which proves that oocytes can serve as a system for structural and binding studies on human proteins due to their possessing a similar environment to that found in human cells [87]. Using this approach, lanthanide-labeled proteins were able to be injected into oocyte. Distance restraints such as PCSs [115] and paramagnetic residual dipolar couplings (RDCs) [128] can be obtained, which can be utilized for determining protein structures and monitoring conformational changes. This method has been successfully used for structural studies on GB1 protein whose folding could be obtained in living cells [69,70].

### 3.4. In-Cell NMR in Insect Cells

**Figure 2:**
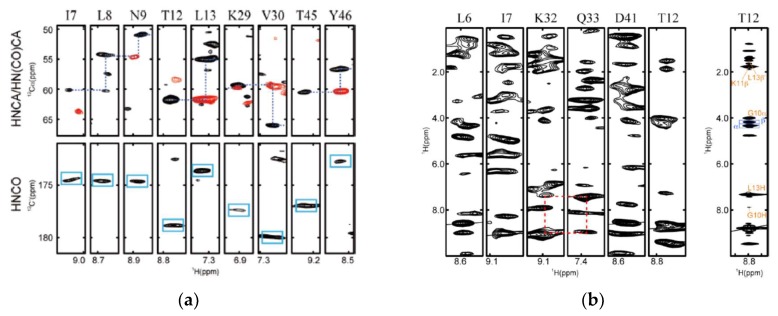
3D NMR spectra collected in sf9 cells. (**a**) 3D HNCOCA (red), HNCA (black) and HNCO spectra of GB1 in sf9 cells. Blue box indicate the C’ signals. (**b**) Selected strip plot at ^1^HN−^1^H dimensions from the 3D a ^15^N-separated NOESY spectrum of GB1 expressed in sf9 cells. The Cα connectivity and sequential NOEs are indicated as blue and red lines, respectively. This figure was reprinted with permission from the reference [71]. Copyright (2013) American Chemical Society. For more experimental details, please refer to the original publication.

The first in-cell NMR study in insect cells was carried out by Hamatsu et al. using GB1, HB8 TTHA1718, rat calmodulin, and human HAH1 as examples [71]. In the study, the target genes were transfected into sf9 cells using a baculovirus system and both ^15^N-and ^13^C/^15^N-labeled proteins were achieved by growing cells in suitable media. In addition to collecting the 2D ^1^H-^15^N-HSQC spectrum, the authors collected 3D triple-resonance NMR spectra that are routinely used in backbone assignment (Figure 2). Approximately 80% of signals from backbone atoms were observed, which made the backbone assignment of GB1 possible. The quality of the acquired 3D ^15^N-seperated NOESY spectrum (Figure 2) was good enough for structural determination as the cross peaks in the spectrum could be assigned [71].

### 3.5. In-Cell NMR in Human Cells

Overexpression and purification of isotopically labeled proteins from mammalian cells for in vitro NMR studies is normally more challenging than in bacteria due to the experimental cost. In-cell NMR in mammalian cells is important for structural studies of mammalian proteins. To carry out in-cell NMR in mammalian cells, researchers have developed different approaches. One outstanding method is to transform isotopically labeled proteins into the cells through a cell-penetrating peptide (CPP), which is derived from HIV-1 tat protein and can be linked with the target protein through fusion or crossing reactions by disulfide bonds. The structures of ubiquitin and FKBP12 were investigated using this approach [93]. There are several types of CPPs that can be used for protein delivery while the conditions need to be explored in the experiments.

In addition to CPP, toxins were used for delivering isotopically labeled proteins into human cells for in-cell NMR studies. Treatment of nonadherent 293F cells with bacterial toxin streptolysin O (SLO) enabled pore formation on the cell membrane. As the diameter of the pores could reach 35 nm, proteins could reach inside of the cells [91]. Supplying Ca^2+^ in the medium was able to prevent cell death caused by pore formation on the cell membrane to reduce releasing of isotopically labeled protein into the medium [91]. Proteins such as isotopically labeled Tβ4 were able to be delivered into the human cells and exhibited dispersed cross peaks in the NMR spectra [91]. Labeled proteins could also be delivered into cells by electroporation, which was originally used for nucleic acids transfection. Modification of Parkinson’s disease protein alpha-synuclein was monitored using in-cell NMR [94,95]. Fusing the target protein with a suitable sequence can localize the protein to desired cellular compartment [67,129], which makes it possible to monitor protein structures in the natural compartments.

In-cell NMR studies in human cells are also achievable using cells with overexpressed proteins. The existing strategies for protein expression in mammalian cells are suitable for producing isotopically labeled proteins for NMR studies [107]. A detail protocol has been developed to produce proteins in mammalian cell lines such as human embryonic kidney 293T (HEK293T) for in-cell NMR studies. In this method, the gene encoding for the target protein is induced into the cells using transient DNA transfection. Isotope-enriched protein is then produced by growing the cells in a medium with ^15^N-nitrogen sources [116]. This method has advantages over protein delivery, as the target protein is produced directly into the living cells, without any protein purification procedures [107]. Using human SOD1 as an example, the metal binding and effect of copper binding on the redox state of the protein were investigated in the living cells [75]. Folding of Mia40 controlled by cytoplasmic glutaredoxin 1 and thioredoxin 1 was evaluated using in-cell NMR [99]. Mia40 was shown to be stable in the cytoplasm. Such studies provide a view of protein folding in living cells at an atomic level, which is challenging to investigate using other biophysical methods [99]. As the expressed proteins can be translocated to certain sub-cellular compartments, protein structure and folding at certain organelles can be evaluated using solution NMR spectroscopy. Folding of Mia40 and hSOD1 was studied on the intact mitochondria using solution NMR spectroscopy. In addition to proteins, the folding of DNA can also be studied using in-cell NMR. The structure of the DNA i-motif was observed in Hela cells using NMR [101] and the obtained information is useful for future biosensor development.

## 4. In-Cell NMR in Probing Protein–Protein Interactions

In-cell NMR provides an ideal system to probe protein–protein interactions, as proteins do not exist as a single molecule under the physiological conditions [130]. To probe protein–protein interactions in bacterial cells, the target protein is normally first overexpressed in a M9 medium to achieve isotope labeling (^15^N). Then the cells were transferred to a normal medium. The binding partner is sequentially induced using another inducer to achieve overexpression. With the extension of induction time, the amount of the binding partner is increased, which is similar to the titration experiment in vitro [131]. Using such a sequential protein expression system, in-cell NMR was used to probe protein–protein interactions in *E. coli* Rosetta (DE3) cells [80]. This study was used to probe the interaction between ubiquitin and proteins with ubiquitin interacting motif (UIM), namely ataxin 3 protein (AUIM) and the signal-transducing adaptor molecule STAM2 [80]. This study provides a unique view of protein–protein interactions in live cells [80].

The number of amino acids that are involved in the molecule interactions might not be correctly estimated in in-cell NMR experiments, as signal broadening is also associated with the formation of stoichiometric complexes in the living cells. To overcome the shortcoming brought about by conventional analysis of the data, Single Value Decomposition (SVD) was proposed to analyze the in-cell NMR binding data [131]. SVD is a mathematical method that can be used to identify the principal components from an arbitrary matrix that was built up from experimental data. SVD has wide applications, and it has been used to process NMR spectra, to determine ligand binding site using information derived from chemical shift perturbations, and to identify allosteric binding sites [132,133,134]. This method was used to analyze the interactions between the prokaryotic ubiquitin-like protein and mycobacterial proteasome ATPase (Mpa) in living cells [135]. Thioredoxin was shown to have exchanges with other cell components and exhibited a molecular weight of approximately 1 MDa in the living cells. In addition to probing protein–protein interactions, an in-cell NMR study showed that adenylate kinase (ADK) had an open binding pocket binding to ATP and AMP [81]. Human PFN1’s specific and unspecific interactions with other proteins were analyzed using in-cell NMR [82]. Accumulated studies have proven that in-cell NMR provides a new avenue to understand protein regulation in the living cells [68].

## 5. In-Cell NMR in Drug Discovery

In-cell NMR has been shown to be used in different cells, giving rise to the possibility of exploring folding and modification [89] of proteins in physiological environments. Probing protein and drug interactions in living cells is critical in drug discovery, as this information is helpful for medicinal chemists to improve the potency of the compounds. As the interactions are monitored in living cells, it is very helpful to understand the action mode of the developed compounds. Monitoring protein and ligand interactions using in-cell NMR has been successfully carried out in living cells by Banci and Hasnain’s team. In their studies, SOD1 was confirmed to form a complex with ebselen, which is an organoselenium compound with broad antioxidant properties [98]. Oxidation of SOD1 in living cells by ebselen was investigated using in-cell NMR. Ebselen was shown to interact with SOD1 and affect its folding in the living cells. This study provides a potential therapeutic application by indicating an unusual SOD1 disulfide bond [98].

### 5.1. Application of In-Cell NMR in Ligand Screening

Protein and ligand interactions can be demonstrated in living cells by monitoring the signals from the substrate. The enzymatic activity of new Delhi metallo-b-lactamase subclass 1 (NDM-1) expressed in *E. coli* cells can be assayed by monitoring the signals from its substrate meropenem [76]. The inhibition of NDM-1 by inhibitors can be monitored using a ^1^H-based experiment. This study provides a direct view of the function and inhibition of enzymes in living cells [76]. A similar strategy could also be applied to human cells when the target in drug discovery is from a human being. The NMR spectra of the development compound in the absence and presence of human cells with and without expressed target protein will prove whether the compound binds to the target protein in living cells. Such studies could also be improved to provide more information by incubating compound with human cells harboring different types of target proteins such as mutations. ^19^F-NMR spectroscopy is also very powerful in in-cell NMR studies, as the background signals from the living cells are reduced because the biological system does not contain fluorine atoms. Cleavage of the fluorinated anandamide analog-ARN1203 was observed in the presence of HEK293 cells harboring expressed fatty acid amide hydrolase (FAAH) [77]. As FAAH is a membrane protein, the assay is feasible using this system, and compound fragments which were able to inhibit its activity were screened and confirmed [136]. The molecular interactions between Bcl-2 and the quercetin-alanine bioconjugate were investigated using proton-based NMR experiments [104]. This study shows that ligand-based NMR such as STD is also applicable in in-cell NMR.

**Figure 3:**
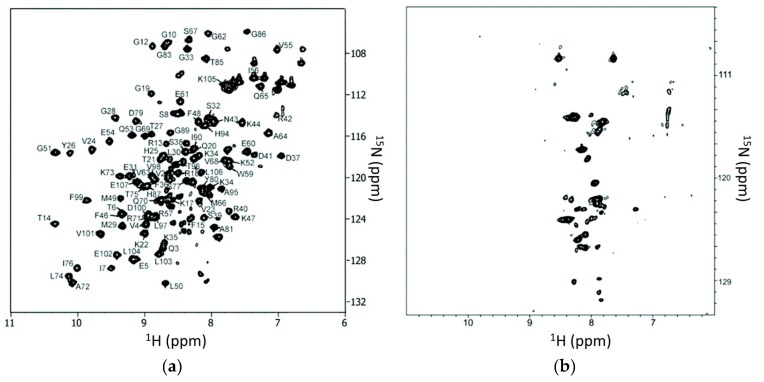
Application of in-cell NMR in compound screening. Peptides that can disrupt FKBP12 and FRB interactions were screened using this approach. (**a**) ^1^H-^15^N-HSQC spectrum of FKBP12-FRB complex in which FKBP12 is ^15^N-labeled. (**b**) Peptide affects the spectrum of FKBP12-FRB complex. This figure was reprinted with permission from the reference [84]. Copyright (2009) American Chemical Society. For more experimental details, please refer to the original publication.

Screening of compounds capable of disrupting protein–protein interactions is feasible using in-cell NMR [84,137]. A system comprising FK506 binding protein 12 (FKBP12) and the 100-residue FKBP-rapamycin binding domain from the mammalian target of rapamycin (FRB) was used in the study. Uniformly ^15^N-labeled FKBP12 and unlabeled FRB were expressed in *E. coli* using a co-expression system. The complex exhibited a ^1^H-^15^N-HSQC spectrum with nicely dispersed cross peaks. Adding rapamycin (binding to FKBP12 with 200 pM affinity) to the solution induced chemical shift perturbations for both FKBP12 and FRB while adding ascomycin to the cell solution induce changes the spectrum of FKBP12 but not FRB, which might be caused by their slightly different binding surfaces on FKBP12. As the existence of two proteins is required to generate the detectable in-cell NMR spectra, this system was then used for screening against a peptide library (Figure 3). Peptides able to disrupt FKBP12 and FRB interactions were identified from a library with 289 dipeptides. The screened peptides were confirmed to disrupt protein–protein interactions in yeast [84] by means of competition experiments with rapamycin and ascomycin. Using a similar method, small molecular compounds that can affect Pup and Mpa interactions were screened from a library consisting of 1597 compounds [83]. To reduce the time for screening, the developed matrix method in which the library compounds were placed a matrix plate and mixed was proven to be a practical and efficient strategy [84].

### 5.2. Application of In-Cell NMR in Target Engagement

Target engagement is a procedure to evaluate protein and ligand interactions in living cells [138]. It is important to understand the molecular action of the developed compounds in drug discovery. As the cellular environment is different from in vitro biochemical environments, the ligand-binding information obtained in vitro might be different from that obtained in vivo. As developed compounds need to be tested in different animal models and different cell lines before they enter into clinical studies, target engagement is therefore critical, as it can provide the real-time binding information in living cells. Several methods have been used in target engagement such as cellular thermal shift assay [139,140] and polarized microscopy [141]. In-cell NMR is a unique tool to study protein–ligand interactions in living cells, suggesting that it can be used as a tool in target engagement [142]. The successful application of in-cell NMR in compound screening and its feasibility for incorporation with other cellular-based mythologies [143,144] make it possible for it to be applied in target engagement.

In-cell NMR was used to validate target engagement of the antituberculosis imidazopyridine amide (IPA) series in living cells [117]. This study used ligand-observed ^1^H and STDexperiments to confirm drug binding to the cytochrome b in living cells. In addition, the atoms of IPA that are important for interactions were also identified in the binding study, which was helpful for obtaining the structure of the complex. The authors used a heterologous host *M. smegmatis*—a non-pathogenic bacterial system—to avoid the handling of pathogenic bacteria in the NMR spectrometer. This is the first application of an in-cell NMR study in target engagement, and it was encouraging with respect to the possibility of carrying out similar investigations in drug discovery.

## 6. Perspective

Most structures deposited in the protein data bank are obtained under in vitro conditions, which might differ from those obtained in living cells, as only purified proteins are used in structural determination in vitro. Proteins under national conditions interact with multiple proteins, which cannot be monitored using in vitro structural methods. In-cell NMR will connect the available structures to protein function in vivo. As in-cell NMR studies can be carried out in prokaryotic and eukaryotic cells, cell biology techniques are required to carry out successful in-cell NMR experiments.

In the drug discovery process, probing protein–drug interactions is a critical step in target-based drug discovery. In-cell NMR is therefore a powerful method to evaluate potent compounds in drug development to save experimental cost. The in-cell NMR study in bacteria will be helpful in antibiotic development as both target engagement and compound transportation into the cells can be monitored. It has been noted that some pathogenic bacteria might not be allowed in NMR studies. The in-cell experiments in mammalian cell lines will be critical both for monitoring protein post modifications and target engagement in developing chemotherapies against human diseases, such as anti-cancer drugs. With the development of new NMR hardware, new methods in sample preparation, and combination with other techniques, in-cell NMR will play more important roles in structural biology and drug discovery.

## Figures and Tables

**Table 1:** Some types of experiment used in in-cell NMR studies ^a^.

Experiment	Remarks	Reference
^1^H-^15^N-HSQC (heteronuclear single quantum coherence)	Protein–protein/ligand interactions	[66,67]
3D experiments	Backbone assignment	[68]
PCS (pseudo-contact shift)	Protein structure determination using lanthanide tags	[69,70]
NOESY (Nuclear Overhauser effect spectroscopy)	Protein structure determination	[71]
SOFAST-HMQC (Band-Selective Optimized Flip Angle Short Transient- heteronuclear multiple quantum coherence)	Protein–protein/ligand interactions	[72]
^1^H-^13^C HSQC	Protein structure analysis using selectively protonation and ^13^C labeling	[68]
^19^F-NMR	In-cell protein-observed ^19^F can be obtained	[73]
Relaxation	Protein dynamics	[74]
Residue dipolar couplings	Lanthanide tags can also be used to generate RDCs	[69]
Protein-based-^1^H NMR	^1^H-NMR at His residue regions	[75]
Ligand-based ^1^H NMR	Protein-ligand interactions	[76]
^19^F-NMR	Ligand observed ^19^F-NMR was used in ligand binding studies	[77]

^a^ Not all the references are listed in the table for the same type of experiments.

**Table 2:** In-cell NMR studies of proteins in different cells.

Cells	Targets	Studies	Reference
Bacteria	TTHA1718	Structure was determined in the living cells	[68]
	calmodulin, NmerA, and FKBP (FK506 binding protein)	Labeling methyl groups of protein was used in-cell NMR studies	[78]
	HdeA, alpha-synuclein, chymotrypsin inhibitor 2 (CI2) ubiquitin	Protein dynamics in cells, protein leakage, and protein–protein interactions were analyzed	[63,79,80]
	Thioredoxin	Quandary interactions of proteins in cells was addressed in the study	[81]
	ADK (adenosine kinase)		
	FKBP		
	Alpha-synuclein, ubiquitin, HDH (histidinol dehydrogensase), GFP (Green fluorescence protein)	Protein-based ^19^F-NMR study was carried out	[73]
	SOD1 SOD1 (human copper, zinc superoxide dismutase 1)	Protein folding in living cells was analyzed.	[72]
	PFN1 (protein profilin 1)	Protein–protein interaction was studied in living cells	[82]
	Pup (prokaryotic ubiquitin like protein)	In-cell NMR was used to screen compounds disrupting protein–protein interactions	[83]
	Mpa (mycobacterial protease ATPase)		
	FKBP12	In-cell NMR was used to screen a library.	[84]
	Cox17 (cytochrome c oxidase copper chaperone)	In-cell NMR was used to probe protein folding in living cells	[85]
oocyte	Ubiquitin, calmodulin	Protein–protein interactions were probed in oocyte	[86]
	GB1 (the B domain of G protein)	Structural studies were performed using PRE restrains	[70,87,88]
	XT-GB1 (SV40 regulatory domain-GB1)	Protein phosphorylation was monitored in cells	[89]
yeast	Ubiquitin	Structural studies were carried out in cell compartments	[90]
Insect	GB1, HB8 TTHA1718, rat calmodulin, and human HAH1	3D experiments were collected in living insect cells for structural studies.	[71]
Mammalian cells	Tβ4 (thymosin β4)	Introducing proteins into cells using toxin was used for in-cell NMR studies.	[91]
	Thioredoxin	Redox status of intracellular thioredoxin was measured in living cells	[92]
	GB1	Labeled protein was delivered into mammalian cells using peptides for in-cell NMR	[93]
	FKBP12		
	Alpha-synuclein	Protein modification and folding were monitored	[94,95]
	hSOD1 and mutants	Folding in living cells and protein–protein interactions were analyzed	[96,97]
	SOD1	Effect of ebselen and ebsulphur on protein structure was investigated	[98]
	Mia40 (mitochondrial intermembrane space import and assembly protein 40)	Protein folding in living cells was investigated	[99]
	Cox17	Protein folding was investigated in living cells	[100]
	DNA i-motif	Stability of DNA i-motif was investigated.	[101]
	copper binding protein HAH1	Sequential protein expression in mammalian cells and selective labeling proteins was used in-cell NMR studies	[102]
	DJ1	Protein folding was investigated	[103]
	Bcl-2 (B-cell lymphoma 2)	Protein-ligand interactions. Saturation-Transfer Difference (STD) and TrNOE experiments were carried out	[104]
	PFN1	Specific and unspecific interactions in cells was explored using in-cell NMR	[82]

**Table 3:** Some representative in-cell NMR studies.

System	Experimental Outcome	Reference
*E. coli*	Heteronuclear spectra of proteins were collected in living cells	[66]
*E. coli*	Protein structure was determined in living cells	[68]
Mammalian cells	In-cell NMR study of proteins that were delivered into cells was performed	[93]
Oocyte	Lanthanide tag was used in generating distance restraints in living cells	[115]
HEK293T	Protein was overexpressed in mammalian cells for in-cell NMR studies	[116]
*E. coli*	In-cell NMR was used to screening a library	[84]
*M. smegmatis*	The first application of in-cell NMR in target engagement	[117]
*Hela*	In-cell NMR study on DNA was carried out	[101]

## Abbreviations

NMR	Nuclear magnetic resonance
Cryo-EM	cryogenic electron microscopy
STD	Saturation-transfer difference
SOFAST-HMBC	Band-Selective Optimized Flip Angle Short Transient-heteronuclear multiple quantum coherence)
Mia40	Mitochondrial intermembrane space import and assembly protein 40
NOESY	nuclear Overhauser effect spectroscopy)
XT-GB1	SV40 regulatory domain-GB1
Cot17	cytochrome c oxidase copper chaperone
Tβ4	thymosin β4
Bcl-2	B-cell lymphoma 2
GB1	the B domain of G protein
HSQC	heteronuclear single quantum coherence
UIM	ubiquitin interacting motif
hSOD1	human copper, zinc superoxide dismutase 1
SVD	Single Value Decomposition
ADK	adenylate kinase
FBDD	Fragment-based drug discovery
CPP	cell-penetrating peptide
PCS	pseudo-contact shifts
RDC	Residue dipolar coupling
PFN1	human protein profilin 1
FKBP12	FK506 binding protein 12
FRB	the 100-residue FKBP-rapamycin binding domain from the mammalian target of rapamycin
Pup	prokaryotic ubiquitin like protein
Mpa	mycobacterial protease ATPase
HDH	Histidinol Dehydrogensase
GFP	Green Fluorescence Protein
